# PP2A_1_ Binding, Cell Transducing and Apoptotic Properties of Vpr_77–92_: A New Functional Domain of HIV-1 Vpr Proteins

**DOI:** 10.1371/journal.pone.0013760

**Published:** 2010-11-01

**Authors:** Angélique N. Godet, Julien Guergnon, Amélie Croset, Xavier Cayla, Pierre Barthélemy Falanga, Jean-Hervé Colle, Alphonse Garcia

**Affiliations:** 1 Laboratoire E3 Phosphatases, Unité Signalisation Moléculaire et Activation Cellulaire, Institut Pasteur, Paris, France; 2 Physiologie de la Reproduction et des Comportements, INRA CNRS UMR 6175, Université de Tours, Haras Nationaux, IFR 135, Nouzilly, France; 3 Unité de Biologie des Populations Lymphocytaires, CNRS-URA 1961, Institut Pasteur, Paris, France; University Hospital Zurich, Switzerland

## Abstract

**Background:**

The hallmark of HIV-1 pathogenesis is the progressive CD4^+^ T cell depletion and high propensity of CD4^+^ T cells to apoptosis. HIV-1 viral protein R (Vpr) is a major pro-apoptotic gene product. A first Vpr-mediated apoptotic mechanism that requires a physical interaction of HIV-1 Vpr_71-82_ mitochondriotoxic domain containing the conserved sequence _71-_HFRIGCRHSRIG_-82_ with the Adenine Nucleotide Translocator (ANT) has been characterized. The family of Ser/Thr protein phosphatase PP2A interacts with several viral proteins to regulate cell growth and apoptotic pathways. Previous studies based on yeast two hybrid assays and mutational experiments indicated that PP2A_1_ is involved in the induction of G2 arrest by HIV-1 Vpr.

**Principal Findings:**

Experiments combining pull-down, cell penetration and apoptosis analyses in distinct human cells indicate that the PP2A_1_ binding sequence from Vpr_77–92_ is a new cell penetrating apoptotic sequence. We also found that the I84P mutation or the IIQ/VTR_83–85_ and T89A substitutions in the Vpr_77–92_ sequence prevent PP2A_1_ binding, cell penetration and apoptosis. In addition the double R77A and R80A mutation known to inactivate the mitochondriotoxic Vpr_71–82_ domain, has no effect on the biological properties of the Vpr_77–92_ domain.

**Conclusion:**

Together our data provide evidence for the first time that the Vpr_77–92_ sequence delineates a biological active domain of Vpr with PP2A_1_ binding and pro-apopototic capacities and, it is conceivable that this cell penetrating sequence may account for the Vpr internalization in uninfected cells. Finally, our data also implicate the existence of two partially overlapping pro-apoptotic domains in the Vpr C-terminal part, a redundancy that represents a new approach to address the question of biological relevance of HIV-1 Vpr. In this context, future studies will be required to determine the functional relevance of the Vpr_77–92_ domain in full length Vpr protein and also in entire HIV provirus.

## Introduction

HIV infection leads to the depletion of CD4+ T cells in patients. The CD4+ T cells depletion of non-infected CD4+ T cells has been correlated to an increased propensity to apoptosis, which relays on induced host and viral factors [1]. HIV-1 Viral Protein R (Vpr) is one of the regulatory HIV-1 proteins that are important for *in vivo* establishment and/or maintenance of AIDS pathogenesis. Multiple studies indicate that Vpr regulates viral replication *in vivo* and is required for virus replication in non-dividing cells. In addition, Vpr induces cell cycle arrest in proliferating cells, stimulates virus transcription and can induce apoptosis of infected cells [2].

Previous studies also suggested that Vpr can induce apoptosis in distinct human cells as a consequence of the prolonged cell cycle arrest [3], [4]. Other reports have clearly documented a major apoptotic mechanism which is based on the physical interaction of Vpr with the Adenine Nucleotide Translocator (ANT), a component of the permeability transition pore of mitochondria localized in the inner mitochondrial membrane [5], [6]. This mitochondriotoxic domain contains Vpr_71–82_ sequence that is partially located at the end of the third α-helix of Vpr (Vpr_55–77_). Vpr is actively secreted when it is produced during the end part of the virus cycle and has been found in biological fluids of patients. Interestingly it is capable of permeating uninfected cells and may be responsible for bystander effect.

The reversible phosphorylation of proteins controlled by protein kinases and protein phosphatases is a major mechanism that regulates a wide variety of cellular processes. Protein phosphatase type 2A (PP2A) represents a major family of serine/threonine protein phosphatases that has been implicated in the regulation of many cellular events, including cell growth and apoptosis in mammalian cells [7]. PP2A proteins comprise dimeric or heterotrimeric enzymes. The dimeric PP2A core enzyme consists of a catalytic C subunit (PP2Ac) and a structural A subunit. A third subunit (B, B', B” or B” ') can eventually bind to the core and regulate both the substrate specificity and localization of the trimeric holoenzyme. PP2A_1_ is a trimeric form composed of A, Bα and C subunit [8]. To specifically compete with the binding of regulatory subunits to PP2A core or to PP1 catalytic subunit, we previously described a novel approach named Drug Phosphatase Technology (DPT). This approach is based on the use of penetrating peptide sequences that interact with PP1/PP2A holoenzymes to specifically disrupt or modulate apoptotic pathways [9].

In this study, we first identify 89.6-Vpr_77–92_ sequence as a PP2A_1_ binding domain. In addition we showed that IIQ/VTR_83–85_ and T89A substitutions in the pNL4.3-Vpr_77–92_ sequence, inhibits PP2A_1_ binding and apoptosis. These results suggest that PP2A_1_-binding site of Vpr_77–92_ C-terminal sequences control the cell penetrating death activities of this new functional domain. Altogether, these data allow us to define DPT-Vpr_1_, a new cell penetrating death molecule, derived from the sequence of 89.6-Vpr_77–92_.

## Results

### Vpr_72–92_ is a PP2A_1_ binding sequence

**Figure 1:**
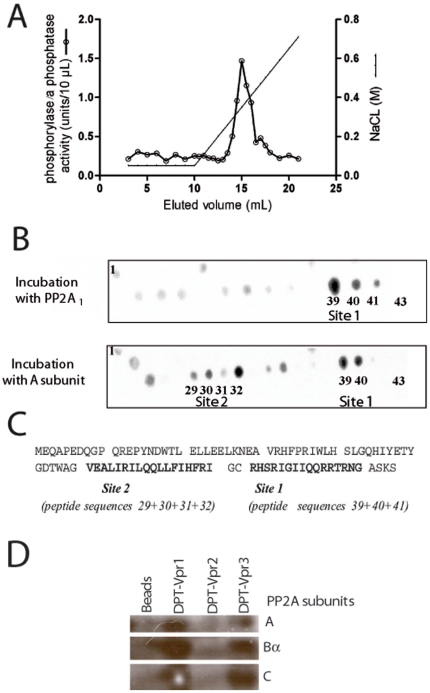
Analysis of HIV-1 Vpr and PP2A1 interaction. (A) 16 µg of PP2A1 holoenzyme purified from pig brain was loaded on a 1 mL HIV-1 strain 89.6-Vpr-T-agarose column equilibrated in buffer C (25 mM TrisHCl pH 7.4, 50 mM NaCl, 0.1 mM EDTA). The column was washed with 10 mL of buffer C and then developed with a 18 mL linear gradient of buffer C to buffer C plus 1 M NaCl. Phosphatase activity of collected fractions was assayed with phosphorylase a as substrat (1U  = 1 pm of phosphate liberated by 10 µL of fraction/min). A major peak of phosphatase activity was detected, eluting at 0.3 M NaCl. (B) ECL revelation of PP2A binding assay on cellulose-bound Vpr peptides. 43 overlapping dodecapeptides with a two-amino acid shift scanning the entire 89.6-Vpr sequence (accession number: AAA81039) were synthesized on a cellulose membrane. The membrane was incubated with PP2A1 holoenzyme (upper panel) or PP2A-A subunit (lower panel) and subsequently with anti-PP2A antibodies followed by peroxydase-labelled anti-guinea pig antibodies. (C) Sequence of HIV-1 Vpr protein: sequence of the two PP2A-A binding sites are indicated in bold. These sequences correspond to overlapping sequences of positive dodecapeptides 29 to 32 (PP2A-A subunit incubation) or 39 to 41 (PP2A1 incubation). (D) Co-precipitation of DPT-Vpr peptides with PP2A1 in HeLa cell extracts. Control magnetic beads coupled with streptavidin alone or conjugated with DPT-Vpr peptides at a concentration of 100 µM, were incubated with cells extracts (5.105 cells). Identification of bound proteins in pull-down experiments was realized by immunoblotting using antibodies against PP2A1 subunits A, Bα and C.

Since previous experiments have indicated that the cell PP2A_1_ holoenzyme is involved in the mode of action of Vpr, we explored whether Vpr and PP2A_1_ could establish physical interaction by performing affinity chromatography. Recombinant full length Vpr-T protein encoded by the HIV-1 (89.6 isolate) was coupled with column agarose beads and purified PP2A_1_ holoenzyme was chromatographied on the column. Phosphorylase *a* phosphatase activity was found exclusively in the fractions eluted by salinity gradient and picking at 0.3M NaCl (Fig. 1A). In addition, the phosphatase activity from both eluted Vpr-Agarose column and from purified PP2A_1_, display a similar sensitivity to protamine stimulation indicating the conservation of PP2A_1_ subunit composition and enzymatic properties after Vpr-agarose binding (Fig.S1). Furtheremore, to identify Vpr sequences able to bind *in vitro* to PP2A_1_, a series of 43 overlapping dodecapeptides from 89.6-Vpr were synthesized onto a cellulose membrane. The membrane was incubated with purified PP2A_1_ holoenzyme or with isolated PP2A_1_ subunits. Three overlapping peptides corresponding to a docking site, named site 1, that binds to trimeric PP2A_1_ holoenzyme were detected (Fig. 1B). Similarly two overlapping peptides corresponding to the same PP2A_1_ docking site 1 were also detected when the membrane was incubated with the structural PP2A-A subunit (Fig. 1B). In addition, we also detected four overlapping peptides that define a second putative binding site, named site 2, for PP2A-A subunit. In contrast, no interactive spot was detected with purified PP2A-Bα and PP2Ac subunits (data not shown). These data allowed to identify RHSRIGIIQQRRTRNG (Fig.1C) as a new Vpr sequence that binds *in vitro* to the PP2A_1_ holoenzyme through its structural PP2A-A subunit.

### The 89.6-Vpr_77–92_ peptide sequence interacts with PP2A_1_ in cell extracts

We further characterized the capacity of Vpr_77–92_ domain to bind PP2A_1_. By using streptavidine magnetic beads, we attempted the PP2A_1_ co-precipitation following incubation of extracts from a variety of cell lines with biotinylated peptides relaying to the Vpr_77–92_ the docking site 1 sequence. The sequence alignments of Vpr proteins and the different peptides tested are respectively listed in Table 1 and Table 2. Both DPT-Vpr_1_ and DPT-Vpr_3_, that respectively correspond to the wild type and a mutated Vpr_77–92_ sequence of 89.6 viral isolate, pulled down PP2A_1_ in cell extracts. DPT-Vpr_3_ varied from DPT-Vpr_1_ by the replacement of three residues by a residue A at the positions R72, R75 and T89. In contrast the IIQ/VTR_83–85_ substitutions or the single residue replacement (I84P) corresponding to DPT-Vpr_2_ and Vpr_4_ peptides respectively prevented detection of any PP2A_1_ subunit in the pull down samples (Fig. 1D, Fig. S2 and Fig. S3, and data not shown). Taken together the pull down experiments account for the specific binding of DPT-Vpr_1_ peptide to PP2A_1_. Our results also suggest, that in addition to the residue I84, the other residues in vicinity (residues 83–85) play a critical role for the targeting the PP2A_1_-binding domain. Future genetic analyses will be required to adress their contribution.

**Table 1:** Sequence alignment of Vpr proteins from two different origins.

ORIGIN	SEQUENCES
	***HELIX I*** ***HELIX II***
	1 10 20 30 40 50
89.6	MEQAPEDQGPQREPYNDWTLELLEELKNEAVRHFPRIWLHSLGQHIYETY
NL4.3	MEQAPEDQGPQREPYNEWTLELLEELKSEAVRHFPRIWLHNLGQHIYETY
	60 70 80 90
	***HELIX III***
89.6	GDTWTGVEALIRILQQLLFI *HFRIGC* *RHSRIG* IIQQRRTRNG ASKS
NL4.3	GDTWTGVEALIRILQQLLFI *HFRIGCRHSRIG* **VTR** QRR **A** RNGAS **R**S
	*** *

Single letter code for amino acids (aa) is used. The three α-helixes are Helix I, aa 17–33, Helix II, aa 38–50 and Helix III, aa 56–77. Variable sequence of the PP2A_1_ binding domain (77–92) is in red. Non variable sequence of mitochondrial death domain is in italic (71–82). *Asterisks indicate distinct residues in 89.6-Vpr (accession number: AAA81039) and Vpr_pNL4.3_ (accession number: AF324493) that are located in PP2A_1_ binding domain. Mutational studies in yeast, suggested that the T89A substitution in NL4.3-Vpr has no apoptotic effect [46]. The K95R substitution in 89.6-Vpr is outside of PP2A_1_-death domain.

**Table 2:** Origin of peptides containing wild type and mutated Vpr sequences derived from 89.6 and pNL4.3 sequences used in this study.

ORIGIN	ACRONYM	SEQUENCES
89.6	DPT-Vpr_1_	RHSRIGIIQQRRTRNG
NL4.3	DPT-Vpr_2_	RHSRIG **VTRQRR**A**RNG**
89.6	DPT-Vpr_3_	**A** HS **A** IGIIQQRR **A** RNG
89.6	DPT-Vpr_4_	RHSRIGI **P** QQRRTRNG
NL4.3	*Vpr_71–96_	HFRIGCRHSRIG **VTR** QRR **A** RNGASRS

-Mutated residues in DPT-Vpr_3_/DPT-Vpr_4_ peptides containing Vpr_77–92_ sequences derived from 89.6 and divergent residues from pNL4.3 in DPT-Vpr_3_ and in *Vpr_71–96_ are in bold.

-*From Arunagiri et al.^11^

### The Vpr_77–92_ sequence from HIV-1 89.6 strain is a new cell penetrating domain

**Figure 2:**
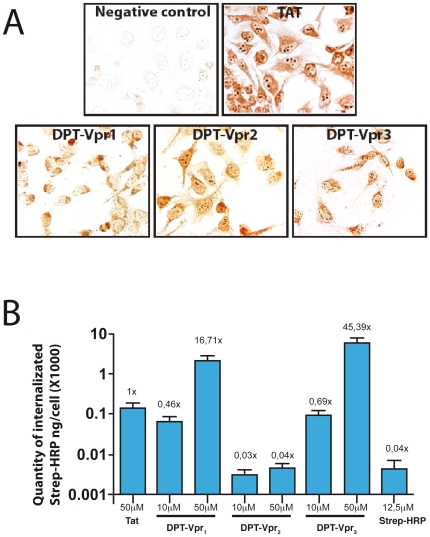
Effect of DPT-Vpr peptides on cell penetration and intracellular delivery of streptavidin-peroxydase. (A) For penetration and localization analysis, cells were incubated with 150 µM of peptides for 2 h at 37°C. After fixation, the presence of biotinynlated peptides is revealed by incubation of permeabilized cells with streptavidin-peroxydase. The sequence of non penetrating peptide used as negative control is GVIFYLRDK. The sequence of positive Tat control is YGRKKRRQRR. (B) Intracellular delivery of streptavidin-peroxydase by biotinylated-DPT-Vpr and Tat peptides in HeLa cells. Streptavidin-peroxydase coupled with biotinylated peptides were incubated for 6 h at 37°C and internalized complexes were visualized by a colorimetric test. Statistical analysis was carried out using Anova's test and significance was assessed at p<0.0001.

It is well established that Tat peptides derived from the cell penetrating protein HIV-1 Tat are cell penetrating sequences that allow intracellular delivery of proteins. Tat transduction depends on various factors, and current protocols permit the transduction into many cells [10]. Since Vpr is a known as cell penetrating protein, we hypothesized that DPT-Vpr peptides containing the Vpr_77–92_ sequence could also display a transduction activity. To test whether DPT-Vpr peptides display similar activity, biotinylated DPT-Vpr peptides were assessed to penetrate and to deliver a protein marker (Streptavidin-HRP) into HeLa cells. As shown in figure 2A, Tat, DPT-Vpr_1_, DPT-Vpr_2_ and DPT-Vpr_3_ peptides penetrate into HeLa cells. However, Tat and DPT-Vpr peptides showed subtle cell staining differences concerning their intra-cellular localization. While Tat, DPT-Vpr_1_ and DPT-Vpr_3_ mostly provided a homogeneous cytoplasm labeling, DPT-Vpr_2_ provided also nuclear/nucleolar labeling. Then the capacity of the biotinylated DPT-Vpr peptides to cargo protein into cell was analyzed. The amounts of HRP internalized by incubating the cells for five hours with three different biotinylated DPT-Vpr Streptavidin-HRP complexes were shown in figure 2B. DPT-Vpr_1_ and DPT-Vpr_3_ peptides (50 µM) resulted in higher levels of HRP internalization than Tat-peptide, the positive reference of the assay (16.7 and 45.3 fold increase respectively). Although DPT-Vpr_2_, was found able to permeate cells (Fig. 2A), surprisingly no cargo effect could be detected with cells incubated with Streptavidin-HRP linked to biotinylated-DPT-Vpr_2_, the signal recorded remained as low as the cells control incubated with the media plus Streptavidin-HRP alone. Thus the lack of cargo effect with DPT-Vpr_2_ peptide correlated with its inability of binding to PP2A_1_ from cell extracts.

### The Vpr_77–92_ sequence from HIV-1 89.6 strain is a new cell death domain

**Figure 3:**
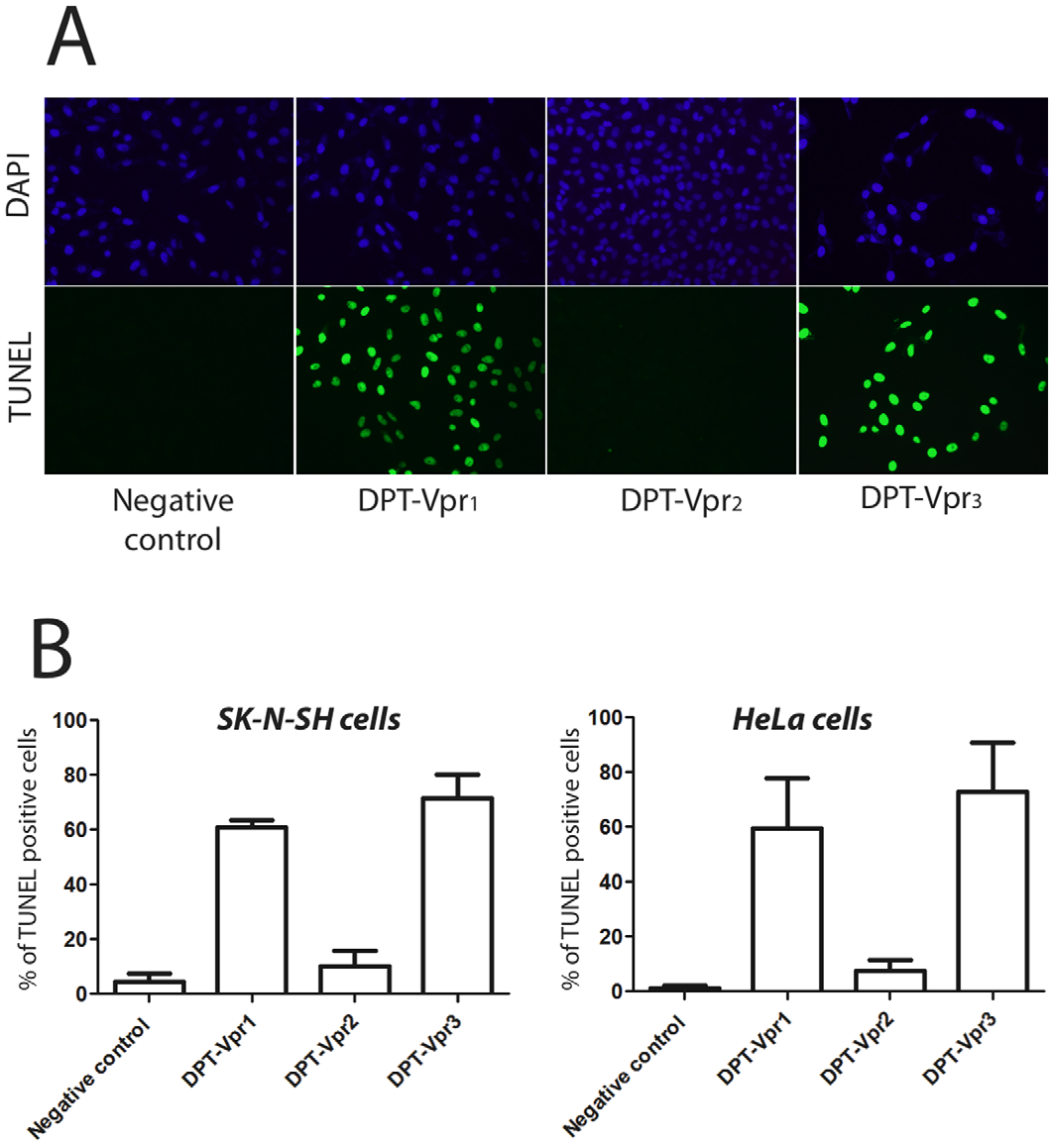
Effect of DPT-Vpr peptides on cell death. (A) Nuclear morphology illustrated with SK-N-SH cells was evaluated with DAPI (blue) and DNA strand breaks were identified using TUNEL labeling (green). (B) Histograms show percentages of positive SK-N-SH cells (left panel) or HeLa cells (right panel) detected in TUNEL assay.

**Figure 4:**
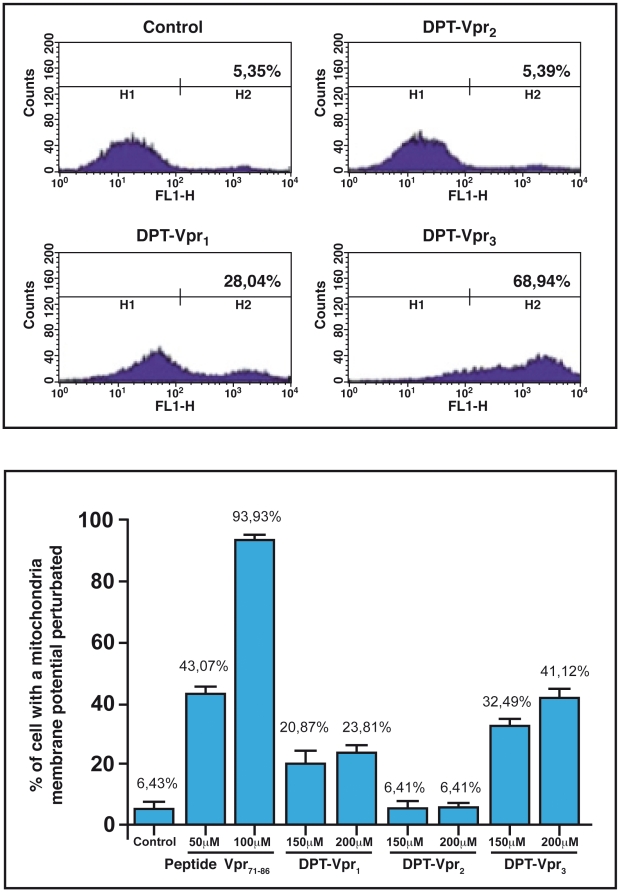
To monitor apoptosis in Jurkat cells treated with DPT-Vpr peptides, we used Annexin V (upper panel) or DiOC6 (lower panel) assays. For DiOC6 statistical analysis was by Anova and significance was set at p<0.05.

Given the critical role of PP2A in the control of apoptotic events and in consistence with the rational of our DPT concept, we hypothesized that DPT-Vpr_1_ could contain a naturally occurring pro-apoptotic DPT-sequence. To test this possibility, we first used TUNEL assay to monitor apoptosis in adherent HeLa or neuronal SK-N-SH cells. Figure 3A illustrates the high apoptotic level obtained in SK-N-SH cells treated with DPT-Vpr_1_ and DPT-Vpr_3_, in contrast, DPT-Vpr_2_ has no significant effect. This apoptotic effect was quantified in both Hela and SK-N-SH cells and we found that DPT-Vpr_1_ and DPT-Vpr_3_ induce apoptosis in 60 to 75% of the cells (Fig. 3B). DPT-Vpr_1_ and DPT-Vpr_3_-meditated apoptosis was also assayed in non-adherent Jurkat T cells using Annexin V, an early marker of apoptosis. As indicated in figure 4 upper panel, DPT-Vpr_1_ and DPT-Vpr_3_ peptides but not DPT-Vpr_2_, induced phosphatidylserine translocation. Furthermore we also analyzed a possible drop in mitochondrial membrane potential (ΔΨm), an earlier event in apoptosis. We found that similarly to Vpr_71–96_, a control peptide containing the mitochondrial death domain from pNL4.3 [11], DPT-Vpr_1_ and DPT-Vpr_3_ but not DPT-Vpr_2_ induced a decrease in ΔΨm (Fig. 4 lower panel). All together these results indicate that the Vpr_77–92_ sequence from 89.6 isolate is a new PP2A_1_-binding death domain. Interestingly divergent residues with 89.6 Vpr_77–92_ sequence are located in the Vpr_77–92_ domains from a previously published long-term non progressive (LTNP) cohort [12].

## Discussion

Vpr is a small basic protein that seems to be well conserved in HIV-1, HIV-2 and SIV [13]. However the sequence of the non-helical C-terminal portion, Vpr_71–96_, appears to be divergent in HIV-2 and SIVmac and previous results indicate that HIV-1 Vpr proteins contain a specific and highly conserved mitochondrial death domain [14] (see table 3). In this work for the first time we reported on biological activity of Vpr_77–92_, a short sequence located on C-terminal part of Vpr delineated by the residues (77–92). We demonstrated that this sequence is a second apoptotic domain.

**Table 3:** Amino acid sequence alignement of Vpr proteins from different origins in the vicinity of mitochondriotoxic and PP2A-dependent death domains.

ORIGIN	SEQUENCES
HIV-1 89.6-Vpr	_71-_HFRIGC RHSRIG IIQQRRTRNG_-92_
HIV-1 LAI-Vpr	------ ------ ------A---
HIV-1 NL4.3-Vpr	------ ------ VTR---A---
HIV-2 GH1-Vpr	-L-A-- NR---S QTRRR-PFP-
SIVmac-Vpr	---G- I----- QPGGGNPLSA

Vpr_71–92_ sequence from 89.6 isolate contains the two distinct overlapping mitochondriotoxic Vpr_71–82_ and PP2A Vpr_77–92_ death domains. Non-identical residues in HIV-1 (LAI and NL4.3), HIV-2 (GH1) and SIVmac are shown.

### The Vpr_77–92_ sequence is a new multifunctional biological domain with PP2A_1_-binding, cell penetrating and cell death properties

Initial studies based on yeast two hybrid experiments suggested that Vpr interacts with the structural PP2A-A subunit and can induce cell cycle arrest [15], [16]. Furthermore, the role of PP2A in Vpr-induced G_2_ arrest was previously analyzed by pharmacological and genetic analyses in yeast and in mammalian cells [17], [18], [19], [20], [21]. Here, based on biochemical studies we have found that the trimeric PP2A_1_ holoenzyme binds to full length Vpr protein. Using peptide bound assays we clearly found that Vpr can interact with the PP2A-A subunit. Furthermore, we determined two distinct sequences, Vpr_57–73_ named site 2 and Vpr_77–92_ named site 1, that interact with the structural PP2A-A subunit. However, only site 1 is able to bind PP2A_1_ holoenzyme. This may suggest that the A subunit of the trimeric PP2A_1_ holoenzyme could contain a masked docking region for site 2 which appears to be accessible on the isolated PP2A-A subunit. Furthermore since site 2 is located in the third helix of HIV-1 Vpr it is conceivable that this kind of helical structure cannot interact with the trimeric PP2A_1_ holoenzyme. Indeed, this position also corresponds to a Leu/Ile-rich domain (amino acids 60–81), which is critical for dimerization [22] and therefore this domain should be unable to intracellularly interact with PP2A_1_. These observations clearly suggest that site 2 represents a cryptic and possibly a non functional PP2A-binding sequence. Furthermore PP2A_1_ binding specificity of DPT-Vpr_1_, a peptide containing the site 1 sequence, was substantiated by testing several peptides modified by residue substitution. We found that the IIQ/VTR_83–85_ substitutions or the single residue substitution I84P prevented the PP2A_1_ binding. In contrast the double substitution, R77A and R80A, allowed the binding to PP2A_1_ holoenzyme.

According to Drug Phosphatase-Technology concept PP2A interacting sequences could represent new biologically active cell penetrating peptides [9]. It is well establisehed that, similarly to some cationic peptides, Tat peptide derived from the HIV-1 Tat protein possessed protein transduction activity. Compared to the activity of Tat peptide, DPT-Vpr_1_ has a more potent activity in transporting streptavidin-HRP molecules. In contrast the DPT-Vpr_2_ peptide that contains the IQQ/VTR_83–85_ and T89A substitutions is unable to deliver streptavidin-HRP molecules into HeLa cells. This result suggests that intracellular transduction is favored by the specific interaction with PP2A_1_ when peptide is in the cell. A similar situation has been previously observed using PP1, PP2A or PKA interacting sequences coupled to inactive non-transducing shuttle DPT-sh1 [9], [23]. Previous studies showed that the C-terminal moiety of Vpr LAI strain (containing the residues 52–96) possessed transducing activity in human cell lines [24]. Vpr_55–91_ sequence containing our newly identified PP2A_1_ binding domain is the most efficient transducing sequence. In addition, Taguchi *et al* ***.,*** [25] identified a shorter sequence DTWPGVEALIRILQQLLFIH FRIGCQH corresponding to the residues 52–78 of the third α-helix domain of HIV-1 Vpr as a new cell penetrating sequence with transducing activity. It is noteworthy that both Vpr_55–91_ and Vpr_52–78_ respectively contain the *in vitro* PP2A-A binding site 2 and the site 1 sequences identified in this study. In accordance with our published DPT-concept, this observation highlights a potential general role of PP2A in transducing efficiency. Further studies will however be required to establish a possible biological relevance of Vpr site 2 in PP2A-mediated transduction pathways. Previous reports indicate that full length Vpr or peptides with sequences derived from the C terminus Vpr proteins enter the cell and cause death of human cells and yeast cells [5], [11], [26]. This death effect requires the presence of a short C-terminal domain containing the conserved mitochondrial death sequence _71-_HFRIGCRHSRIG_-82_.

### The Vpr_77–92_ domain : a new approach to decipher Vpr-induced apoptosis in human cells

An area of studies has documented the capacity of Vpr to induce apoptosis. Few intracellular Vpr targets that may direct different mechanisms of apoptosis pathways have been identified. Previous reports indicate that Vpr association with a Cul4A-containig E3 ligase complex via its association with DCAF1 prevents ubiquitination and degradation of cellular protein required for cell cycle progression [27]. The possibility that Vpr association with this complex may be a causality link between cell cycle control arrest and apoptosis occurrence has recently garnered credit. Residues distantly spread on Vpr (*Q*3, *Q*65, *R*77, *R*80) have been found to play some role in G2 cycle arrest illustrating the fact that whole part of the protein is required for active cell cycle arrest. Interestingly the R80A substitution renders also Vpr inactive for cell cycle arrest but the R77A and R80A substitutions that affect direct binding of Vpr 71–82 to ANT and inactivate the michondriontoxic domain activity did not prevented apoptosis induction [28]. By testing these substitutions with DPT-Vpr_3_ we did not find obvious impact on the pro apoptotic property of our new Vpr_77–92_ domain suggesting that both ANT and PP2A binding domains regulate Vpr-mediated apoptosis. Indeed ANT and PP2A_1_ binding domains have a seemingly redundant ability to direct an apoptosis process indicating that the Vpr_71–92_ sequence is a new structural and functional domain.

It was previously published that the mutation R77Q of HIV-1 Vpr frequently occurs in HIV-1 infected Long Term Non Progressor (LTNP) patients [12]. This mutation impairs the molecular interaction of HIV-1 Vpr proteins with ANT resulting in inactivation of the mitochondrial Vpr_71–80_ death domain [14]. However this is a controversial observation, more recently challenged by studies reporting that the mutation R77Q can't be associated to reduce HIV-1 infectivity [29]. Interestingly, since Vpr is also present in the body fluids of HIV-1 patients including plasma and CSF [30], [31], it is conceivable that both soluble full length Vpr proteins and C-terminal processed fragments could induce a bystander death effect in non-infected CD4+ cells. We also can hypothesize that Vpr_77–92_ sequence from entire or C-terminal processed protein sharing the penetrating properties of 89.6- Vpr_77–92_ sequence, should penetrate and provoke apoptosis in both infected or non infected cells.

Neurocytopathic effects induced by a recombinant Vpr molecule in neuronal cells suggest that Vpr is involved in the genesis of neurological disorders [32], [33], [34], [35]. In addition, it has been shown that Vpr can be found in brain section of HAND patients, specifically in astrocytes and neurons [36] and neuronal cell death was observed in these patients, concomitantly with the presence of Vpr protein and transcripts [37]. Furthermore, in contrast to peptides containing the mitochondriotoxic sequence (residues 71–82), the peptidic sequences spanning the C-terminal domain of Vpr (residues 52–96 or 70–96) induced apoptosis in cultured rat cortical and striatal neurons [38]. These data imply that a direct interaction between Vpr and ANT alone is inefficient to induce apoptotic pathway in neurons [38], [39]. Interestingly, our cell death experiments in neuron derived SK-N-SH cells indicate that DPT-Vpr_1_ is also potently active in neuronal cells. In consistence with the existence of soluble forms of Vpr in biological fluids, our results likely suggest a biological effect of our new biologically active Vpr_77–92_ domains in Vpr-mediated neurodegeneration.

In conclusion, our identification of a new biological domain has multiple consequences for development of future researches in HIV-1 field. First it is a priority to study whether or not the Vpr_77–92_ domain is biologically active in the context of the full length Vpr protein and of HIV replication. Interestingly, the complex structural organization of the *vpr* gene with two overlapping pro-apoptotic sequences raises several questions about its biological relevance. We can speculate that such gene organization should increase genomic information to counteract the HIV genomic variation occurrence. In addition the existence of overlapping ANT and PP2A_1_ binding sequences may lead to eventual binding competition between the two ANT and PP2A_1_ pro-apoptotic cell targets. Because Vpr is prone to be interacting with an area of cells, such as infected resting and/or activated cells and non-infected cells, it is conceivable that either pathway may concern preferentially alternate cell phenotypes.

In addition our results suggest the hypothesis that non active sequences of the new PP2A_1_-dependant Vpr_77–92_ death domain could be implicated as a regulator of long-term non-progression as well as a viral pathogenicity factor. Especially, functionally active PP2A_1_ binding Vpr_77–92_ death sequences in HIV-1 isolates should be a positive event for AIDS progression and potential development of HIV Associated Dementia (HAD). Focusing on the case of HAD, since some ART treatments don't cross the blood-brain barrier [36], [37], it will be important to identify new DPT-peptides or/and to design new chemical drugs that compete with Vpr_77–92_ PP2A_1_ binding sequence. Such synthetic blockers of the Vpr_77–92_ domain/PP2A_1_ interaction will obviously have an impact on the HIV-1 pathogenesis. More generally, the use of new DPT-peptides targeting PP2A_1_ will be useful to study different neurodegenerative diseases such as Tauopathies in which PP2A_1_ plays an important role [40]. These molecules should have a more specific action than currently available drugs.

## Materials and Methods

### Kits and Reagents

An 8 well Lab-Tek II Chamber Slide, Nalge Nunc International), streptavidin Horse Radish Peroxidase conjugate (Euromedex,), 3,3′-diaminobenzydine tetrahydrochloride (DAB) of DAB Substrate Kit for Peroxidase (Vector laboratories), “complete mini EDTA free” protease inhibitor cocktail from Roche, O-Phenylenediamine-dihydrochloride (OPD) tablet from Sigma chemical, Annexin-V-FITC from Roche, DiOC_6_(3) from Sigma, Kit DeadEnd™ Fluorometric TUNEL system product (Promega), FluorSave™ Reagent from Calbiochem, Vectashield mounting medium for fluorescence, with DAPI (Vector Laboratories), Trypsin EDTA, Invitrogen.

### Cell Culture and Reagents

Adherent HeLa cells (American Type Culture Collection (ATCC): CCL-2, Manassas, VA) were cultured in Dulbecco's modified Eagle's medium (D-MEM, Invitrogen) as exponentially growing sub confluent monolayers in micro plates or in 12-, 24- or 96- well plates. Jurkat lymphoid T cells (clone E6, ATCC: TIB-152) were cultured in RPMI 1640 Glutamax Medium (Gibco; Invitrogen). Both cell lines were cultured in medium supplemented with 10% fetal calf serum (Invitrogen). Adherent SK-N-SH cells (neuroblastoma cells, ATCC: HTB-11) were cultured in DMEM Glutamax-I-High Glucose (Gibco) with 10% fetal calf serum (Gibco/BRL).

### Peptides

High-performance liquid chromatography-purified NH_2_–biotinylated peptides (purchassed from Neosystem) were prepared by solid-phase peptide synthesis. Peptides were dissolved in DMSO and stored at 4°C until use or in half of EtOH and 150 mM NaCl and stored at −20°C pending use.

### Vpr, PP2A proteins and antibodies

Full length HIV-1 89.6-Vpr was prepared in a 6-his C-term tagged form (Vpr-T) by expression in bacteria as described previously [41], [42]. Vpr-T agarose column was generated by binding Vpr-T onto a nickel charged iminodiacetic acid-Sepharose-4B (Amersham Biosciences). Protocol for purification of PP2A proteins: trimeric PP2A_1_ purification from pig brain was adapted from methods previously described [43]. Preparation of recombinant PP2A-A, PP2A-Bα and PP2A-C proteins as well PP2A antibodies (polyclonal guinea pig anti-A, or anti-B and anti-C subunits) has been previously described [44]. In western-blot anti-Guinea Pig-HRP were used to 1/5000 (DAKO P0141).

### *In vitro* phosphatase assay

^32^P-labeled phosphorylase *a* was prepared by phosphorylation of phosphorylase b with phosphorylase kinase and the phosphorylase phosphatase activity was assayed as described previously [43].

### PP2A-binding assays on cellulose-bound Vpr peptides

Overlapping dodecapeptides scanning the whole Vpr sequence were prepared by automated spot synthesis (Abimed, Langerfeld, Germany) onto an amino-derived cellulose membrane, as described [45]. Membrane was blocked using SuperBlock (Pierce), incubated with purified PP2A-A, PP2A-Bα, PP2A-C subunit or PP2A_1_ holoenzyme and after several washing steps, incubated with anti-PP2A antibodies, followed by PO-conjugated secondary antibody. Positive spots corresponding to PP2A-binding peptides were visualized using the ECL system.

### Pull down assays to detect interaction of biotinylated peptides with PP2A_1_ target

Biotinylated peptides were pre-incubated 2 h at 100 µM or 200 µM (in final concentration with lysate) at room temperature with 30 µl of streptavidin-coated immunomagnetic beads (Calbiochem, San Diego CA). During this time, 1×10^6^ HeLa cells at 90% of confluence per peptide point assay were trypsinized, washed with 1 mL of phosphate-buffered saline (PBS), and centrifuged at 600 g and 4°C for 10 min. Cell pellets were lysed 10 min on ice with 400 µl of lysis buffer (50 mM Tris pH 7.4, 200 mM NaCl, 10 mM EDTA, 20% Glycerol, 1% Nonidet P-40, 1 mM phenylmethylsulfonyl fluoride (PMSF), 10 mM NaF, 1 mM orthovanadate, and “complete mini EDTA free” protease inhibitor cocktail from Roche). Lysates were clarified at 13,000 g for 10 min at 4°C and after rotation with supernatant at 4°C for 2 h biotinylated peptides were pulled down with streptavidin magnetic beads and washed twice with 750 µl of lysis buffer. Bound proteins and clarified lysates were analyzed by western blotting using PP2A_1_ antibodies.

### Cellular Localization of biotinylated Peptides

A total of 4×10^4^ exponentially proliferating HeLa cells growing in a sub confluent monolayer were seeded per well on a Lab-Tek and incubated at 37°C, 5% CO_2_. Biotinylated peptides were added 48 h later to the cells at different concentrations for different periods of time. Cells were washed twice in PBS, and fixed with absolute ethanol for 10 min at –20°C before addition of 100 µM of streptavidin HRP conjugate After 30 min incubation at 37°C, cells were rinsed twice in PBS and incubated with DAB for around 10 min. Cells were washed in distilled water and mounted for microscopic examination.

### Quantification of Peptide Internalization

Before incubation, the peptides were pre-incubated for 1 h with streptavidin HRP conjugate in a 4∶1 ratio. Cells at 80% confluence were incubated with different concentrations of peptide in 24-well plates. After 6 h, cells were washed twice in PBS, trypsinized and harvested in 1 ml of PBS and counted. Cells were lysed in 300 µl of 0.1 M Tris pH 7.4, and 0.5% Nonidet P-40 buffer for 10 min on ice. A total of 50 µl of cell lysate was mixed with 50 µl of OPD buffer (51.4 mM Na_2_HPO_4_ and 24.3 mM citric acid) then mixed with 100 µl of OPD solution according to manufacturer's instructions. After 10 to 20 min, the reaction was stopped by adding 100 µl of 1M HCl, and optical density was read at 490 nm.

### TUNEL Assay

TUNEL assays were performed according to manufacturer's instructions. A total of 4×10^4^ HeLa or SK-N-SH cells were seeded per well on a Lab-Tek II and incubated at 37°C, 5% CO_2_, biotinylated peptides were added to the cells 18 h later at different concentrations for different periods of time. Slides were rinsed three times in PBS and mounted with Vectashield containing DAPI. After that the slides were examined by fluorescence microscopy.

### Annexin-V assays and evaluation of mitochondrial membrane potential (ΔΨm)

Jurkat cells (2.5.10^5^ cells in 400 µL of complete RPMI-1640 medium) were incubated at 37°C and 5% CO_2_ with different peptide concentrations. After 3 h, cells were washed in PBS and we used an Annexin-V-FITC to assess outer leaflet phosphatidylserine (PS) exposure in the plasma membrane as a means for the early detection of apoptotic cells. Staining was performed according to the manufacturer's instructions. We also used the cationic Dye DiOC_6_(3), which localizes into intact mitochondria, to measure ΔΨm. DiOC_6_(3) was added at 20 nM for 15 to 30 min at 37°C in the dark. Cells were washed in PBS and immediately analyzed by flow cytometry. A total of 20,000 cells were analyzed in a FACScalibur flow cytometer (BD Biosciences, San Jose, CA).

## Supporting Information

Figure S1 Comparison of the effect of protamine on PP2A1 and on the phosphatase eluted from HIV-1 Vpr-Agarose column. Phosphorylase a phosphatase activities of purified PP2A1 and from the pool of the 4 most active fractions eluted from HIV-1 Vpr-agarose column chromatography were assayed in the presence of increasing concentration of protamine added without pre-incubation. The activity in the absence of protamine was taken as 100%.
(6.02 MB TIF)

Figure S2 Effect of DPT-Vpr4 peptide on PP2A-interaction, cell penetration, and cell death. (A) Cell penetration was analyzed similarly to Fig. 2A in HeLa. (B) Co-precipitation of the DPT-Vpr4 peptide with PP2A in HeLa cell extracts. Experiment was performed similarly to Fig. 1D and the interaction with PP2A1 was analyzed by immunoblotting using antibodies against the regulatory subunit PP2A-Bα. (C) To monitor apoptosis in Jurkat cells treated with DPT-Vpr4 or with positive control Vpr71-96 peptides we used Annexin V (upper panel) or DiOC6 (lower panel) assays as described in Materials and Methods. Statistical analysis was by Anova and significance was set at P<0.05.
(10.48 MB TIF)

Figure S3 Co-precipitation of DPT-Vpr peptides with PP2A subunits in cell extracts. Co-precipitation of DPT-Vpr peptides with PP2A subunits in SK-N-SH (A) or in Jurkat (B). Immunoblotting was analysis was performed using antibodies against the PP2A-Bα in SK-N-SH and PP2A catalytic subunit in HeLa cells.
(6.02 MB TIF)
