# Supplementary figures and images for: PP2A1 Binding, Cell Transducing and Apoptotic Properties of Vpr77–92: A New Functional Domain of HIV-1 Vpr Proteins
Source: PLoS One. 2010 Nov 1;5(11):e13760. doi: 10.1371/journal.pone.0013760 (PMC2967473; doi:10.1371/journal.pone.0013760)

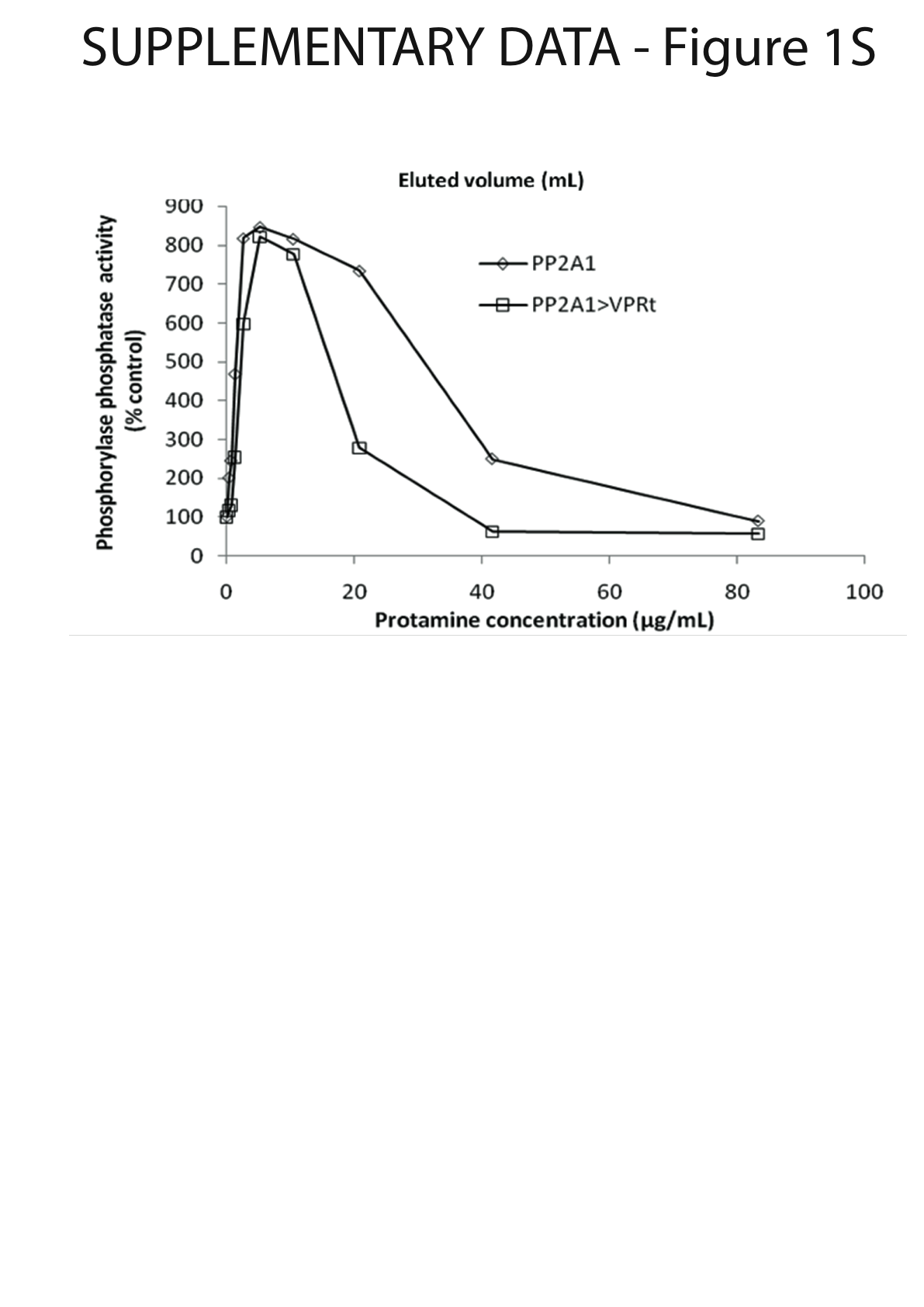

Supplement: Figure S1 — Comparison of the effect of protamine on PP2A1 and on the phosphatase eluted from HIV-1 Vpr-Agarose column. Phosphorylase a phosphatase activities of purified PP2A1 and from the pool of the 4 most active fractions eluted from HIV-1 Vpr-agarose column chromatography were assayed in the presence of increasing concentration of protamine added without pre-incubation. The activity in the absence of protamine was taken as 100%. (6.02 MB TIF) [file pone.0013760.s001.tif]

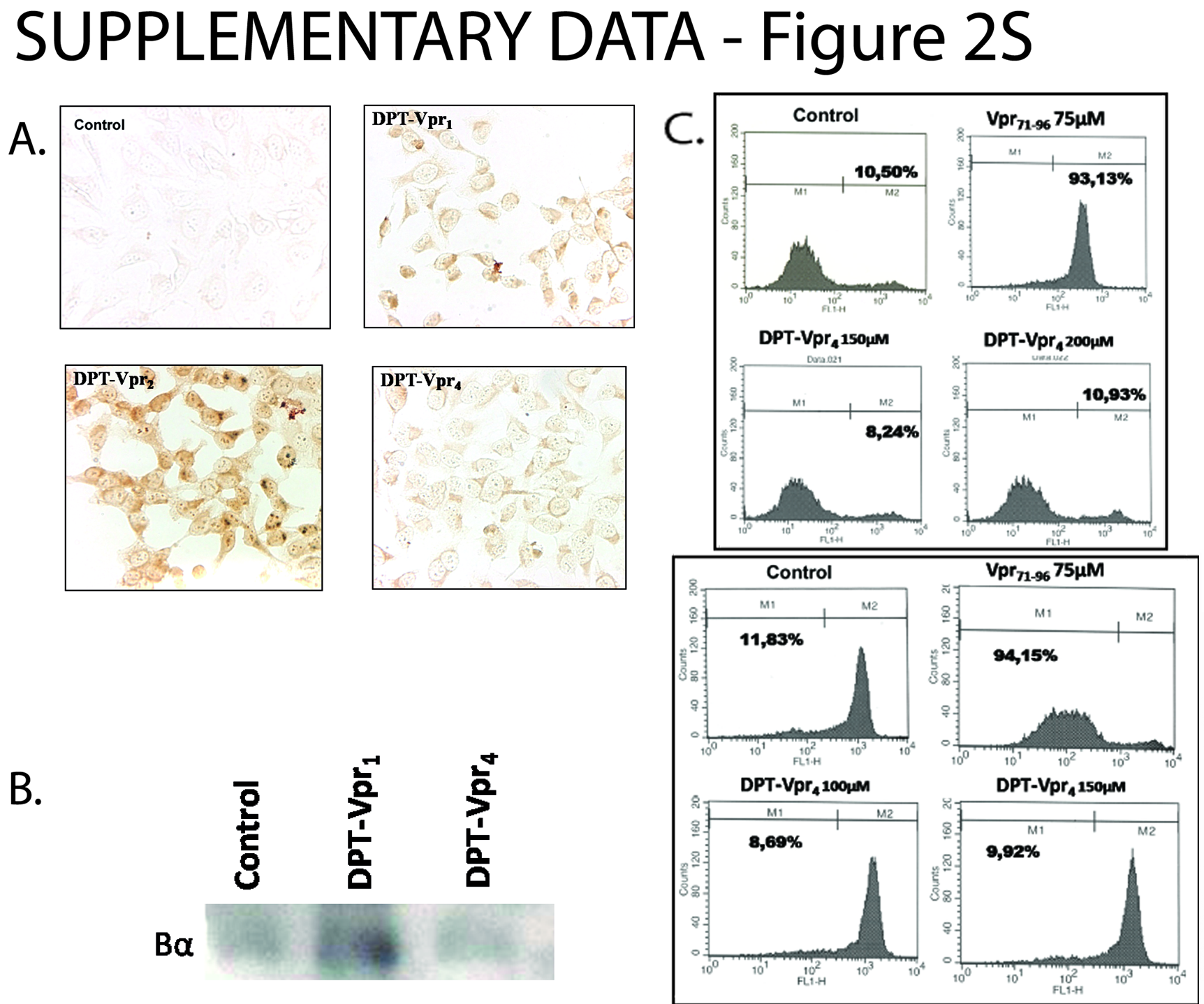

Supplement: Figure S2 — Effect of DPT-Vpr4 peptide on PP2A-interaction, cell penetration, and cell death. (A) Cell penetration was analyzed similarly to Fig. 2A in HeLa. (B) Co-precipitation of the DPT-Vpr4 peptide with PP2A in HeLa cell extracts. Experiment was performed similarly to Fig. 1D and the interaction with PP2A1 was analyzed by immunoblotting using antibodies against the regulatory subunit PP2A-Bα. (C) To monitor apoptosis in Jurkat cells treated with DPT-Vpr4 or with positive control Vpr71-96 peptides we used Annexin V (upper panel) or DiOC6 (lower panel) assays as described in Materials and Methods. Statistical analysis was by Anova and significance was set at P<0.05. (10.48 MB TIF) [file pone.0013760.s002.tif]

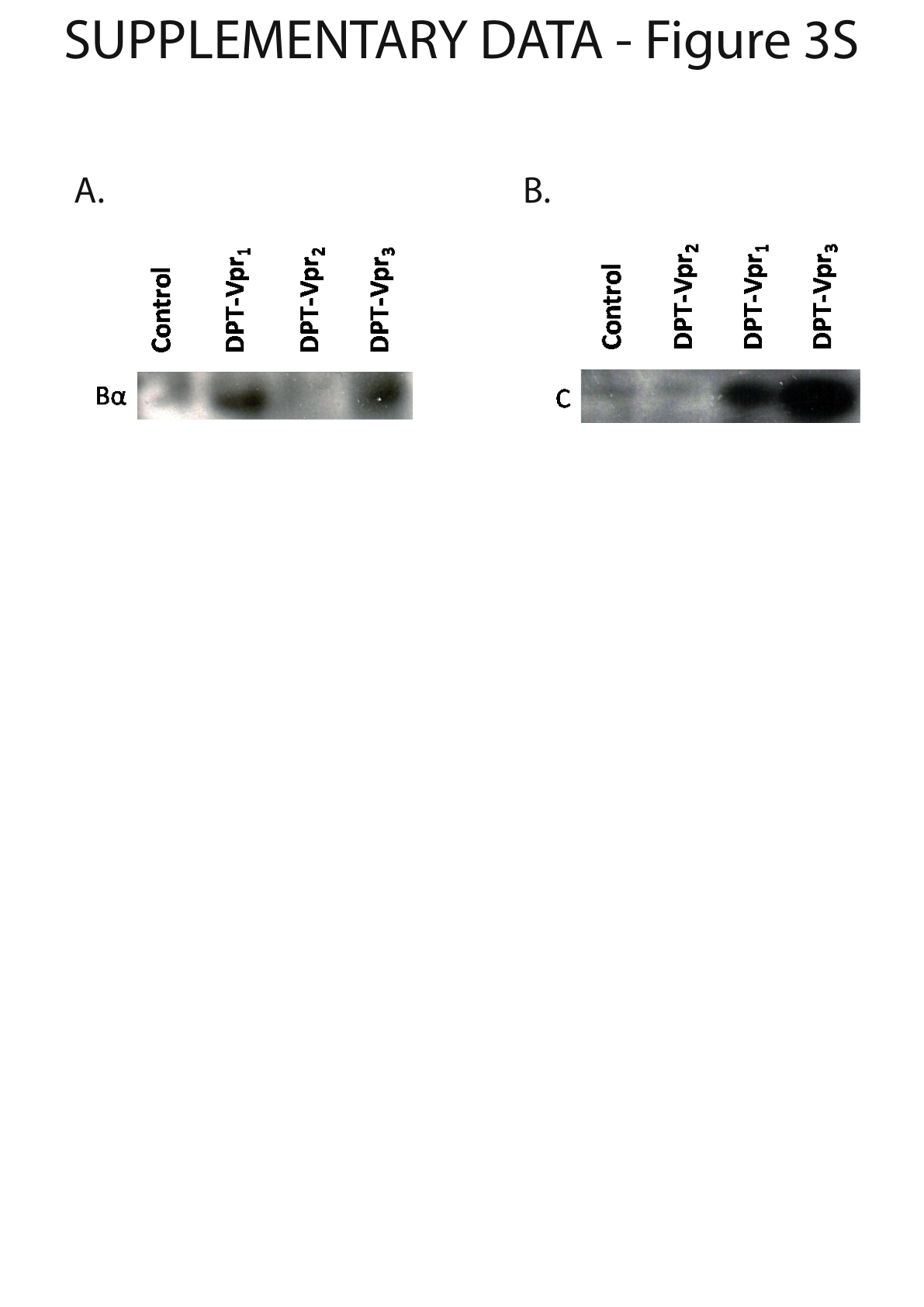

Supplement: Figure S3 — Co-precipitation of DPT-Vpr peptides with PP2A subunits in cell extracts. Co-precipitation of DPT-Vpr peptides with PP2A subunits in SK-N-SH (A) or in Jurkat (B). Immunoblotting was analysis was performed using antibodies against the PP2A-Bα in SK-N-SH and PP2A catalytic subunit in HeLa cells. (6.02 MB TIF) [file pone.0013760.s003.tif]
